# Functional performance tests in male and female professional dancers from four classical ballet companies – a cohort study

**DOI:** 10.1186/s13102-026-01836-0

**Published:** 2026-10-22

**Authors:** Tabea Arens, Anja Hauschild, Martha Richter, Dominik Fohrmann, Astrid Zech, Astrid Junge

**Affiliations:** 1 Institute of Human Movement Science, University of Hamburg, Hamburg, 20148 Germany; 2 Institute of Interdisciplinary Exercise Science and Sports Medicine, MSH Medical School Hamburg, Hamburg, 20457 Germany; 3 Center for Rehabilitation and Sports Medicine, BG Klinikum Hamburg, Hamburg, 20457 Germany; 4 Palucca Hochschule für Tanz Dresden, Dresden, 01277 Germany

**Keywords:** Screening; Functional performance tests, Ballet, Sex differences

## Abstract

**Background:**

Functional performance test (FPT) results have rarely been analysed for sex-specific differences among professional ballet dancers yet. Therefore, this study aims to assess the FPT results of professional female and male ballet dancers, analyse sex-specific differences, describe the number of dancers meeting predefined thresholds, and present the prevalence of lower limb asymmetries in selected FPTs.

**Methods:**

Professional ballet companies in Germany were invited to participate in this study via word-of-mouth. An extensive health & performance screening was provided to the voluntarily participating dancers, which included eight FPTs. Solely dancers on sick leave were excluded. The selected FPTs focused on stability (balance error scoring system (BESS)), muscular endurance & strength (heel-rise test, Bunkie test, and closed kinetic chain upper extremity stability test (CKCUEST)), and hop performance (vertical-hop test, figure-of-eight-hop test, side-hop test, and speedy jump test). Differences in the FPT results between sexes, limb asymmetries in the FPTs (determined by the Limb Symmetry Index (LSI)), and the number of dancers achieving predefined thresholds were analysed with independent-samples *t*-test, and Chi²-tests, respectively.

**Results:**

Data from 115 dancers from four classical ballet companies were included in the analysis. The FPT results differed between sexes: while female dancers performed better than male dancers in the heel-rise test (*p *= .019), males excelled in the CKCUEST (*p*<.001), Bunkie Copenhagen plank (*p *= .004), vertical-hop test (*p*<.001), figure-of-eight-hop test (*p*<.001), and side-hop test (*p*<.001). Lower limb asymmetries greater than 15% were most prevalent in the vertical-hop test (30%) and the side-hop test (24%), and least prevalent in the figure-of-eight-hop test (1%).

**Conclusion:**

This is the first study that analysed sex-specific differences in FPT results of professional ballet dancers from multiple companies. It contributes to the broader understanding of the basic physical performance skills for ballet, presenting the current training status of dancers from four professional ballet companies. The majority of muscular endurance & strength tests and hop performance tests revealed significant differences between male and female dancers, with many dancers showing notable limb asymmetries. Overall, the results demonstrated a lower performance level compared to athletes in other sports.

## Introduction

Ballet dancers, as artistic athletes, require advanced technical skills, great flexibility, well-trained balance and motor control skills, as well as muscular endurance and strength to perform at a high level [1]. Despite the importance of these physical attributes, performance screenings using functional performance tests (FPTs) have rarely been studied in professional ballet dancers.

FPTs serve multiple objectives: they provide an overall impression of the dancer’s performance, identify imbalances in the basic motor skills, dysfunctional movement patterns, and technical deficits [2, 3]. The FPT results can guide tailored training programs aimed at improving identified deficits and imbalances, thereby supporting the long-term health of dancers and maximizing the level of performance [4]. Screenings can be used pre-season, or after injury to assess the dancer’s readiness to return to dance and perform on stage.

There are only a few guidelines or recommendations on how to interpret the screening results of dancers, most of which focus on pre-professionals and university dancers [2, 4–8]. Moreover, the assessments that have been used do not fully represent the broad variety of athletic skills needed for ballet, as they primarily focused on balance, flexibility, and isolated isometric muscle strength [2, 4]. With the use of FPTs more complex movement sequences, which display the necessary integration of the physical capabilities determining performance [9], can be assessed.

Even though it is known that males, for instance, typically exhibit greater absolute and relative muscular strength than females [10, 11], sex-specific differences have not yet been investigated in ballet dancers in the context of FPTs. Presenting and interpreting FPT results by sex could account for the biological differences and distinct performance requirements of ballet between sexes, e.g., in partnering, where males typically lift females [12].

Given the absence of established thresholds for FPTs in professional ballet dancers, it is important to document how dancers perform relative to predefined thresholds from other athletic populations. Understanding how many dancers meet or fall short of these thresholds, derived from literature on other athletic populations [13–15], and consensus in ballet [5], can contribute to future discussions about performance standards and potential injury risk factors.

Dancers are required to have a high degree of symmetry in the upper and lower extremities to realise the creative and physical demands of the choreographer on both sides equally [16, 17]. However, over years of training and practice, most dancers develop a leg preference, particularly for jumps and turns [18]. Additionally, certain choreographies can contribute to the development of asymmetries, and besides that, asymmetries can evolve through injuries and/or chronic pain [19]. The limb symmetry index (LSI) is well-established to determine the extent of discrepancy between the limb’s FPT results, primarily used in return-to-competition decisions [14]. However, limited LSI data is available for professional ballet dancers in hop and strength test results.

Therefore, the primary aim of this study was to analyse FPT data of professional ballet dancers from four companies in a screening battery covering stability, muscular endurance & strength, and hop tasks on sex-specific differences. It was hypothesized that there are differences between male and female professional ballet dancers in FPTs. The secondary aims were to assess lower limb asymmetries, as well as determine the number of dancers whose FPT results met predefined thresholds.

## Methods

### Study design

Between August 2021 and August 2022, professional ballet companies were recruited via word-of-mouth to participate in a comprehensive performance screening. Sample size followed availability. The dancers from the participating companies were informed about the project and provided written informed consent for their voluntary participation. Data collection took place at the beginning of the 2022/23 season (August / September). The screenings were scheduled within the dancers’ working hours, and organized by the company’s management team, though participation remained voluntary. The cohort study has ethical approval (MSH 2021/137) from the MSH Medical School Hamburg, Germany and was performed in accordance with the standards from the Helsinki Declaration. Reporting followed the guidelines of the Strengthening the Reporting of Observational Studies in Epidemiology (STROBE) [20].

### Participants

Four German ballet companies whose management agreed on implementing a health & performance screening at the beginning of the 2022/23 season were included. To participate, dancers had to be full-time employed at one of the participating companies. Exclusion criteria were any current injuries or illnesses resulting in sick leave at the time of the screening. Dancers of both sexes and all age ranges were eligible for inclusion.

### Outcome measures

#### Screening procedure

The FPTs used in this study were selected and partly adapted from standardized protocols used in other sports, such as handball and soccer [14, 21–24]. The FPTs were categorized based on the motor skills assessed: stability (Balance Error Scoring System (BESS)), muscular endurance & strength (heel-rise test, Closed Kinetic Chain Upper Extremity Stability Test (CKCUEST), Bunkie test), and hop tests (vertical-hop test, figure-of-eight-hop test, side-hop test, speedy jump test). All dancers completed the screening in the described order. For all applicable tests, the right leg was assessed first, except for the hop tests, where all tests were performed on the right leg first before transferring the sensor and repeating all hop tests with the left leg. Due to practical reasons, there was no randomization or standardized warm-up.

Screenings were conducted over one to three days, depending on company size. One tester and one assistant screened a group of two dancers, while two to three groups were screened simultaneously. The total testing procedure lasted 75 to 90 min per couple. All tests were demonstrated, explained, and supervised by physiotherapists, physicians, and/or qualified assistants who participated in an extensive training to minimize measurement bias. Since all testers had a dance background, the instructions for the FPTs were provided in technical jargon to ensure optimal understanding. Trials to familiarize with new movement patterns were given. No verbal encouragement was permitted during any of the tests, and no test setup positioned the dancer in front of a mirror to prevent self-correction of posture and alignment.

The BESS test and the heel-rise test were performed barefoot; all other tests were performed in sneakers. All FPTs were performed in the companies’ dance studios, thus on sprung floor, which is designed to create bounce and absorb shock.

#### Functional performance tests

##### Stability test

- Balance Error Scoring System (BESS): This test assessed postural stability under eyes-closed conditions on a foam surface (Airex foam, Airex AG, Inc.) with moderate to good interrater (ICC = 0.57) and intrarater reliability (ICC = 0.74) [25, 26], and fair to good validity (*r *= .42–0.79). It is a validated tool for assessing sport-related concussion injuries with a sensitivity of 34% and specificity of 87% [27], and is commonly utilized for general stability assessments in athletes [28]. Five positions were tested for 20 s each: double-leg stance, single-leg stance (right and left), and tandem stance (right and left) [25]. Hands were placed on the hips. In the single-leg stance, the ankle of the non-supporting leg was held in the coup-de-pied position (pointed foot of the non-supporting leg close to the ankle of the supporting leg, ensuring that the legs did not touch to avoid creating an anchor point). In the tandem stance, both feet were placed behind each other in one straight line, while the toes of the back foot had to touch the heel of the front foot. Errors were counted during each stance and scaled from 1 to 10. An error within the first five seconds of the test automatically resulted in a maximal error score of 10. Errors included loss of balance, touching the ground or the pad with the non-supporting leg or moving it more than 30°, opening eyes, deviating the hands from the hips, lifting both forefeet or heels during tandem stance.

##### Muscular strength & endurance tests

- Heel-rise test: This test assessed single-leg muscular endurance of the calves [29] to the beat of a metronome (60 bpm), a test which has generally demonstrated moderate to excellent test-retest reliability (ICC = 0.57–0.99), and moderate to strong validity (*r *= .56–0.98) [30] in different settings. For this test set-up, one finger of each hand was placed on a bar for balance support and the maximum relevé height was marked by the tester using a hand-held marker which was set at the dancer’s initial maximum relevé. The test was ended when the dancer was unable to reach the marker for two consecutive repetitions, by the dancer themselves, or 30 repetitions were performed.
- Closed Kinetic Chain Upper Extremity Stability Test (CKCUEST). This test assessed the strength and stability of the shoulder girdle in a push-up position with good to excellent reliability (ICC = 0.86–0.95) [31], and strong correlations to the upper extremity star excursion balance test in healthy adults [32]. The feet were placed hip-width apart, and the individual’s arm span (C7 to tip of the longest finger) determined the distance between the tapes on the floor. Dancers tapped over the determined distance, marked by tapes with alternating hands as often as possible within 15 s [33]. Three rounds were performed with a break of 45 s in between. The mean number of correct hand contacts was calculated by subtracting the mean number of error contacts from the mean number of total contacts. Incorrect contacts were defined as failing to reach over the defined lines and tapping higher than the wrist.
- Bunkie test: This test assessed four planking positions to evaluate muscular endurance & strength of full muscular chains, for the right and left side respectively (side plank, Copenhagen plank, front plank and reversed plank) [14, 34].

- Side plank: The dancer performed a plank on one forearm, with the ipsilateral lower leg placed on an approximately 30 cm high stepper and the contralateral leg held in a straight line in the air.
- Copenhagen plank: The dancer performed a side plank on one forearm while squeezing the stepper between both feet. The non-supporting arm was kept on the lateral side of the body during both planking positions. The right side was assessed first before performing both planking positions on the left side.
- Front plank: The body weight was distributed between both forearms and one foot on the stepper, with the non-supporting leg held straight in the air. The right leg was assessed first before shifting the body weight to the left foot on the stepper. The hands were not allowed to be in contact during this planking position.
- Reversed plank: The dancers faced the ceiling while their body weight was distributed between both forearms and one foot on the stepper.
- The time in seconds until the correct position could no longer be maintained, the dancer ended the test by leaving the position, or 46 s were reached for each position and side was recorded with a stopwatch (0–46 s) [14]. Between all positions, a 30 s break was given.

##### Hop tests

For the hop tests, an inertial sensor was worn on the lower leg at the level of the tibial tuberosity (Orthelligent, Oped GmbH, Germany), which was proven to be valid (*r* > .94, *p* < .001) and reliable (intertester reliability (ICC = 0.99; 0.97) and intratester reliability (ICC = 0.96; 0.82)) against a force plate during vertical hop tests [35].

- Vertical-hop test: This test assessed the power of the lower extremity during a single-leg countermovement hop [36] with good to excellent reliability (ICC = 0.82–0.99) [35]. After two submaximal trials, one single-leg hop with maximal effort was performed per leg with the hands placed on the hips. Jump height was measured in cm, conducted by the inertial sensor and calculated by the time of flight. Incorrect attempts, such as moving the arms from the hips, performing multiple hops before achieving a stable landing, or loss of balance, were allowed to be repeated [37].
- Side-hop test: This test assessed lateral stability and endurance of the lower extremity while having dancers hop sideways over two parallel lines for 30 s. A distance of 30 cm was used instead of the common 40 cm in order to decrease fear of (re-)injury during the screening. The original 40 cm side-hop test has excellent validity (ICC = 0.93–1.0) and reliability (ICC = 0.92–1.0) [38]. The number of correct hops was determined by subtracting the error hops (counted by the tester) from the total hops (recorded by the inertial sensor). The criterion for incorrect hops was touching the lines with a part of the foot.
- Figure-of-eight-hop test: This test assessed the speed of consecutive hops with changes of direction [39] with excellent test-retest reliability (ICC = 0.95) [40]. A figure-of-eight is hopped twice around two cones spaced 5 m apart, arms were allowed to swing for speed gain. Starting position was behind a line on the right side of the first cone when testing the right leg, and vice versa for the left leg. The ending position was equal to the starting position. For the timed hop tests (figure-of-eight hop and speedy jump test), the time needed to finish the specific movement task of each test was measured by the sensor. A stable landing was required to stop the timer, with landing time subtracted from the total time. The criteria for incorrect trials included ground contact with the non-supporting foot, incorrect landing position or test execution, not being able to secure a stable landing for two seconds after finishing.
- Speedy jump test: This test assessed power, stability and coordination of the lower extremity while hopping over an obstacle course with forward, backward and sideward movements (TST—Trendsport^®^, Grosshöflein, Austria) with moderate to good test-retest reliability (ICC = 0.79 for the non-dominant leg and 0.83 for the dominant leg) [41]. The time needed for the (most) successful of three trials was recorded by the inertial sensor. The criteria for incorrect trials included touching the bars with the supporting foot, ground contact with the non-supporting foot, incorrect hop order, moving hands from hips, inability to secure a stable landing for two seconds after finishing.

### Data processing & statistical analysis

Data were processed with Microsoft^®^ Excel LTSC MSO (16.0.14332.20587) 64-Bit and analysed with IBM SPSS statistics 28.

Missing data were treated with pairwise exclusion. Data was declared as missing when the dancer reported pain during the testing, and the difference between the dancer’s body sides was ≥ 1 standard deviation (SD). In case of missing data for one side, the mean score was replaced with the available score from the other side.

Thresholds were determined from previous literature and supported by consensus for the categories “below average”, “average”, and “above average” for the following tests:

- Heel-rise test: <20, 20–24, and 25–30 repetitions [5, 42, 43].
- CKCUEST: <21, 21–26, > 26 correct hand contacts [13].
- Bunkie test: <15, 15–29, 30–46 s [14].

The LSI was calculated for the heel-rise test, vertical-hop test, figure-of-eight-hop test, side-hop test, and speedy jump test with an adapted formula:

$$\:LSI=\:100-\left(\frac{limb\:with\:worse\:result}{limb\:with\:better\:result}\right)*100$$

The LSI result was subtracted from 100 to display asymmetry between the legs as a percentage and thus to increase understanding of the dancers. LSI results were grouped into the following categories of asymmetry:

- Low: 0– <11% asymmetry.
- Moderate: 11–15% asymmetry.
- High: >15% asymmetry.

FPT scores were compared between female (F) and male (M) dancers using independent samples *t*-tests. The results are presented as mean ± SD, minimum and maximum values, as well as 95% confidence interval. Differences in the thresholds categories between the sexes were analysed with Chi²-tests, with results presented as percentages. Differences in limb symmetry between the sexes were analysed with Chi^2^-tests, and results are presented as mean ± SD, minimum and maximum values, as well as 95% confidence interval. Alpha level was set on 0.05 for all data to detect significant differences.

## Results

### Demographics

In total, 115 dancers from four professional ballet companies participated in the screening at the beginning of the 2022/23 season. Sex-specific demographics can be found in Table 1. Two companies were considered as large (> 50 employed dancers), one as medium (20 - 50), and one as small (< 20).

**Table 1:** Participant information of this cohort

Participating dancers	Female	Male
	60 (52.2%)	55 (47.8%)
*Age*, *mean ± SD in years*	27.7 ± 6.4	29.1 ± 5.1
*Height*, *mean ± SD in cm*	168.0 ± 5.4	178.7 ± 6.2
*Weight*, *mean ± SD in kg*	52.5 ± 4.9	70.7 ± 5.2
*BMI*, *mean ± SD in kg/m²*	18.6 ± 1.0	22.4 ± 1.3
*Employed at a * *small company*	13 dancers (11.3%)	
*Employed at a * *medium sized company*	31 dancers (27.0%)	
*Employed at a * *large company*	71 dancers (61.7%)	
*Participation rate * *small company*	81.3%	
*Participation rate * *medium sized company*	79.5%	
*Participation rate * *large company*	47.7%	

Large company: > 50 employed dancers, medium company: 20–50 employed dancers, small company: < 20 employed dancers. Participation rate was calculated as the percentage of participating dancers relative to the total number of dancers employed at each company

*SD* Standard deviation

### Sex-specific FPT results of professional ballet dancers

The sex-specific FPT results are presented in Table 2.

**Table 2:** Results of stability & motor control, muscular endurance & strength, and hop tests of female and male dancers

	Female (*n* = 60)			Male (*n* = 55)			Comparison		
**Stability tests**									
BESS, errors (*n*)	*Mean ± SD* *(95% CI)*	*Min.*	*Max.*	*Mean ± SD* *(95% CI)*	*Min.*	*Max.*	*t*	*p*	
* Double-leg stance*	0			0					
* Single-leg stance*	8.2 ± 2.3 (7.5–8.8)	1	10	8.4 ± 2.4 (7.7–9.2)	1	10	- 0.5	0.593	
* Tandem * *stance*	5.4 ± 3.3 (4.6–6.4)	0	10	5.3 ± 3.2 (4.2–6.2)	0	10	0.3	0.759	
**Muscular endurance & strength tests**									
	*Mean ± SD (95% CI)*	*Min.*	*Max.*	*Mean ± SD (95% CI)*	*Min.*	*Max.*	*t*	*P*	
Heel rises, correct repetitions (*n*)	26.8 ± 4.3 (26.0-28.3)	11.5	30	24.7 ± 5.1 (23.1–26.0)	10	30	2.4	0.019*	
CKCUEST, correct contacts (*n*)	17.3 ± 4.4 (16.2–18.6)	8.0	25.3	21.3 ± 4.2 (20.0-22.5)	11.7	30.3	-4.9	< 0.001*	
Bunkie, time (sec.)									
* Side plank*	43.9 ± 5.0 (45.2–42.5)	24.5	46	43.4 ± 6.0 (41.9–45.1)	22.5	46.0	0.5	0.634	
* Copenhagen plank*	27.5 ± 12.4 (31.2–24.4)	5.0	46	33.4 ± 8.5 (31.3–36.1)	10.0	46.0	-2.9	0.004*	
* Front plank*	41.5 ± 7.5 (43.8–40.2)	15.0	46	41.1 ± 6.9 (38.8–42.9)	20.5	46.0	0.2	0.809	
* Reversed plank*	37.4 ± 10.6 (41.2–35.9)	13.0	46	34.5 ± 10.5 (31.0-37.1)	10.5	46.0	1.5	0.136	
**Hop tests**									
	*Mean ± SD* *(95% CI)*	*Min.*	*Max.*	*Mean ± SD* *(95% CI)*	*Min.*	*Max.*	*t*	*p*	
Vertical-hop, jump height (cm)	12.6 ± 2.1 (12.0-13.2)	8.0	16.5	18.5 ± 3.5 (17.5–19.5)	10.5	26.5	-11.0	< 0.001*	
Figure-of-eight-hop, time (sec.)	13.5 ± 1.4 (13.8–13.0)	17.3	11.0	11.9 ± 1.3 (11.4–12.1)	15.1	9.5	6.3	< 0.001*	
Side-hop, correct hops (*n*)	47.4 ± 8.8 (44.8–49.6)	22.5	63.0	54.9 ± 10.5 (51.7–58.0)	26.0	83.5	-4.1	< 0.001*	
Speedy jump, time (sec.)	8.5 ± 0.8 (8.7–8.2)	10.7	7.0	8.2 ± 1.1 (7.9–8.5)	12.5	6.2	1.7	0.100	

*BESS* Balance Error Scoring System, *CKCUEST* Closed Kinetic Chain Upper Extremity Stability Test, *SD* Standard deviation, *CI* Confidence interval, *min.* minimum, *max.* maximum, *t* T-test test statistic

*Statistically significant results with an alpha level of 0.05

In the stability test (BESS), dancers had more errors in the single-leg stance (8.3 ± 2.3, 95% CI: 7.9–8.7) compared to the tandem stance (5.4 ± 3.2, 95% CI: 4.8–6.0), without a significant difference between sexes. In the double-leg stance of the BESS, no errors were recorded for any dancer.

In the muscular endurance & strength tests, the dancers completed an average of 25.8 ± 4.8 (95% CI: 24.9–26.7) heel-rises, and 19.3 ± 4.7 (95% CI: 18.4–20.2) correct hand contacts in the CKCUEST. The dancers maintained a stable posture in the four positions of the Bunkie test as follows: side plank 43.6 ± 5.4 (95% CI: 42.6–44.6) seconds, Copenhagen plank 30.3 ± 11.0 (95% CI: 28.3–32.3) seconds, front plank 41.3 ± 7.2 (95% CI: 40.0–42.6) seconds, reversed plank 36.0 ± 10.6 (95% CI: 34.1–38.0) seconds. There were large inter-individual differences, especially in the Copenhagen plank, where a ninefold difference existed between the least and most proficient outcomes (5.0 vs. 46.0 s). Female dancers significantly outperformed males in the heel-rise test, while males outperformed females in the CKCUEST and the Copenhagen plank of the Bunkie test.

In the hop tests, a jump height of 15.5 ± 4.1 (95% CI: 14.7–16.2) cm was achieved in the single-leg vertical-hop test, 51.0 ± 10.3 (95% CI: 49.0–52.9) correct hops in the side-hop test, 12.7 ± 1.6 (95% CI: 12.4–13.0) seconds in the figure-of-eight-hop test, and 8.3 ± 0.99 (95% CI: 8.2–8.5) seconds in the speedy jump test. The side-hop test showed the widest range of results among the dancers, with correct hops ranging from 22.5 to 83.5 correct hops. Males significantly outperformed females in the vertical-hop test, the figure-of-eight-hop test, and the side-hop test (Table 2).

### Threshold analysis

The results of the threshold analysis are presented in Table 3. The number of dancers not meeting the specific threshold and thus being categorized as below average varied substantially between tests, with the highest number observed in the CKCUEST for both females and males. The distribution of dancers categorized as below average, average, or above average differed significantly between sexes in the heel-rise test, the CKCUEST, and the Copenhagen plank of the Bunkie test.

**Table 3:** Number and percentage of female and male dancers performing below average, average or above average in four Functional Performance Tests

Functional performance tests		Female			Male			Comparison	
		Below average	Average	Above average	Below average	Average	Above average	X²	*p*
Heel-rises	**Thresh-olds**	***< 20***	***20–24***	***25–30***	***< 20***	***20–24***	***25–30***		
	Right leg	4 (6.7%)	13 (21.7%)	43 (71.7%)	8 (14.5%)	20 (36.4%)	27 (49.1%)	6.27	0.044*
	Left leg	1 (6.8%)	11 (18.6%)	44 (74.6%)	6 (11.5%)	20 (38.5%)	26 (50%)	7.23	0.027
CKCUEST correct contacts	**Thresh-olds**	***< 21***	***21–26***	***> 26***	***< 21***	***21–26***	***> 26***		
		44 (74.6%)	15 (25.4%)	0	21 (38.2%)	31 (56.4%)	3 (5.4%)	16.6	< 0.001*
Bunkie	**Thresh-olds**	***< 15***	***15–29***	***30–46***	***< 15***	***15–29***	***30–46***		
*Side * *plank*		2 (3.3%)	9 (15.0%)	49 (81.7%)	4 (7.3%)	6 (10.9%)	45 (91.8%)	1.2	0.543
*Copenhagen plank*		34 (57.7%)	16 (27.1%)	9 (15.3%)	19 (34.5%)	30 (54.5%)	6 (10.9%)	11.6	0.009*
*Front * *plank*		8 (13.3%)	15 (25.0%)	37 (61.7%)	6 (11.3%)	15 (28.3%)	32 (60.4%)	0.215	0.868
*Reversed * *plank*		13 (21.6%)	19 (31.7%)	28 (46.7%)	16 (29.7%)	23 (42.6%)	15 (27.8%)	5.0	0.175

*CKCUEST* Closed kinetic chain upper extremity stability test

*Statistically significant results with an alpha level of 0.05, x² = Chi² test statistic

### Limb symmetry

The results of the lower limb symmetry analysis are presented in Table 4. The LSI values of the dancers showed high variability across the heel-rise test, vertical-hop test, figure-of-eight-hop test, side-hop test, and speedy jump test. The highest number of dancers with a high asymmetry was found in the vertical-hop test and the side-hop test, whereas dancers showed the most symmetrical results in the figure-of-eight-hop test. No statistically significant differences in LSI were found between sexes in any of the five tests.

**Table 4:** Lower limb symmetry index (LSI) of the dancers in five functional performance tests

Functional performance tests	*N*	Mean LSI± SD (95% CI)	Maximum LSI	LSI, *n* (%)		
				High (> 15%)	Moderate (11–15%)	Low (0–<11%)
Heel-rises	111	4.4 ± 6.3 (5.6–3.2)	23.3%	11 (9.9%)	9 (8.1%)	91 (82.0%)
Vertical-hop	111	13.7 ± 14.9 (10.9–16.5)	66.7%	33 (29.7%)	11 (9.9%)	67 (60.4%)
Figure-of-eight-hop	109	5.3 ± 4.0 (6.1–4.6)	19.0%	1 (0.9%)	11 (10.1%)	97 (89.0%)
Side-hop	103	11.0 ± 8.0 (12.6–9.5)	35.5%	25 (24.3%)	20 (19.4%)	58 (56.3%)
Speedy jump	91	7.0 ± 5.2 (8.1–5.9)	26.8%	10 (11.0%)	5 (5.5%)	76 (83.5%)

*LSI* Limb symmetry index, *SD* Standard deviation, *CI *Confidence interval

## Discussion

This study was one of the first to analyse functional performance in professional male and female ballet dancers from multiple German opera houses and state theatres. One of the main findings is that the stability test (BESS) was similar between sexes, while the results of the muscular endurance & strength tests, as well as the hop performance tests, differed between male and female dancers. We also observed a wide range of performance in some parameters, indicating deficits in functional fitness. The highest percentage of dancers performing below average was found in the CKCUEST and Copenhagen plank. The highest percentage of dancers with a high leg asymmetry (> 15%) were observed in the vertical-hop test (29.7%) and the side-hop test (24.3%).

Few studies have investigated FPT results in dancers, with most focusing on pre-professional dancers, flexibility, and movement control [2, 4, 7]. Further, there is no consensus which skills should be assessed, and which tests used yet. The present study implemented a set of eight FPTs in four ballet companies, with the aim to cover the broad spectrum of motor skills determining performance to present comparative values for future performance screenings.

### Sex-specific differences in FPTs results of professional ballet dancers

#### Stability test

In the BESS test, the double-leg-stance emerged as a stable position in this study for both sexes, questioning whether it challenges coordinative skills accordingly and thus its relevance in assessing postural stability in dancers [44]. During tandem and single-leg stance, this cohort of ballet dancers had fewer errors than non-athletes [45], but more than figure skaters [46]. This may be due to the limited transferability of coordinative skills. The BESS creates an unfamiliar setting for dancers using a foam surface and inhibiting visual feedback. In contrast to the figure-skaters, dancers particularly enhance their visual feedback through mirrors, which are present in most studios.

There were no sex-specific differences in this cohort of ballet dancers in the BESS test. This aligns with previous research by Janura et al. [44], which found no effect of sex on centre of pressure data in three standing tasks on firm and foam surfaces, as well as after ten turns in professional ballet dancers. Sex-specific differences in the BESS test were previously reported for healthy adolescents, with girls having better results than boys [47]. However, this difference between sexes seems to neutralize with age, as no sex-specific differences were found in collegiate athletes [48].

#### Muscular endurance & strength tests

Many dancers in this cohort showed lower overall levels of muscular endurance & strength compared to athletes in football and handball [14, 21], consistent with previous studies [29, 49, 50]. The normative values of maximal heel-rises for averagely active adults were higher than the present cohort of dancers was able to perform [51]. This might be caused by ending the test after 30 repetitions in this study’s cohort to prevent muscle soreness from the screening. Since 63% of the dancers in this cohort performed repetitions close to the requested 30 repetitions, a ceiling effect is possible. However, the greater range of motion in the ankle plantarflexion of ballet dancers might require more calf muscle work during the heel-rise [50]. Comparing sexes, females exhibited superior calf muscle endurance. While on average 75% of females exceeded the threshold of 24 repetitions [5], only 49% of males did so. Clinically, it is speculated that this may be attributed to the demands of pointe work. However, the hypothesis that dancers who perform pointe work have greater muscular endurance of the plantarflexors compared to those who do not has yet to be scientifically confirmed.

The results of the Bunkie test indicated varying levels of difficulty across the four planking positions. In this cohort, the longest mean holding times were found in the front and side planks. In contrast, healthy adults, tend to hold the reversed plank the longest [34]. This discrepancy might be caused by the hyperextension of the knees that many dancers face [52], which demands more muscular work to stabilize compared to non-hyperextendable joints [53, 54]. The better results in the side plank may be attributed to the high volume of single-leg work in ballet, particularly during développés, which requires great hip muscle strength in the standing leg, especially in the abductors [55].

The results of the Copenhagen plank indicate a strength deficit in the adductors in ballet dancers. Additionally, the side plank and front plank are well-known exercises that are regularly implemented into the dancers’ personal training, while the Copenhagen and reversed plank are less familiar, which might result in the lower strength adaptations displayed in the latter two tests. Sex-specific differences were found only in the most challenging position, the Copenhagen plank, possibly caused by a ceiling effect in the other planking positions.

The difference between sexes in the CKCUEST emerged despite adapting the distance of the tapes according to the individual arm span, thereby equalizing the level of difficulty for shorter females [33]. This is presumably caused by the focus of training on the lower extremity in ballet, while the upper extremity is mainly targeted in lifting and carrying for male dancers.

#### Hop tests

Hop tests provide valuable insight into the dynamic capabilities of dancers, including power, speed, and coordination of the lower extremity [56]. In this cohort, the dancers had comparable results to healthy sports students in the single-leg vertical-hop test and the speedy jump test [37]. Male dancers, however, demonstrated lower jump heights in the vertical-hop test than non-elite handball players [57], despite ballet emphasizing high jumps and thus a long time of flight to showcase the dancers technical abilities in the air [58]. It should be noted that different measurement devices were used, which may limit the comparability of the results [59].

The relatively low performance in the hop tests among this cohort of ballet dancers may be attributed to the limited stimulus classical dance training provides for musculoskeletal adaptations. Even though beyond the scope of this paper, dancers may not achieve the same musculoskeletal adaptations as athletes who implement targeted training designed to enhance explosive power and endurance. On the contrary, the repetitive demands of high jump count during dance training and performance, as observed by Maloney et al. [60], may lead to fatigue without providing the progressive adaptations needed for peak performance.

Ballet dancers perform a substantial volume of jumps per class (62 to 270 jumps), which exceeds the jumping volume of a basketball match play [60], and face more hours dancing per week than athletes in most other sports spend in training and competition [61]. Yet, about two-thirds of the jumps in a class remain below 50% of the dancers’ maximum countermovement jump height [60].

Unlike state-of-the-art athletic training regimens, the implementation of strategies such as periodization, structured and progressive overload, and supplemental strength training for an optimal improvement of physical capacities are uncommon in dance [61, 62]. On the contrary, the (almost) daily class follows a historical tradition and is not designed to improve maximal output in e.g., jump height. It has been shown that the jumping height of adolescent dancers did not improve when they participated exclusively in dance classes [63]. With additional strength exercises and plyometric jumping tasks, jumping height increased significantly over a 16-week period [61].

In this cohort, male dancers had better test results than female dancers in the vertical-hop test, the figure-of-eight-hop test, and the side-hop test, indicating greater explosive power and better lateral stability in male dancers. This is in line with a previous study in which the countermovement jump height was analysed in adolescent ballet dancers [64]. Furthermore, it has been found that male dancers of a professional ballet company perform a higher count of jumps compared to their female counterparts per class (153 vs. 119 jumps) [60]. No significant differences between sexes were observed in the speedy jump test, possibly due to its greater emphasis on coordination compared to the other hop tests.

### Threshold analysis

The standard threshold of the single-leg heel-rise test is 25 repetitions in professional dancers [5, 43] as well as in other healthy athletes [42]. When performing the heel-rise test with the right leg, 50.9% of the male ballet dancers and 28.3% of the female ballet dancers failed to reach 25 repetitions (respectively 50% of the male dancers and 25.4% of the female dancers when performing the test with the left leg). Dancers are particularly prone to chronic overuse injuries, especially of the achilles tendon [65]. Therefore, ensuring that their achilles tendons can endure the repetitive relevés executed during class, rehearsal and performance is crucial. However, aside from the mentioned consensus [5], there are no published studies linking greater plantar flexor strength endurance to a reduced risk of chronic achilles tendon issues or ankle sprains in dancers [66].

The thresholds for the CKCUEST and Bunkie test were transferred from manuals for football and handball players [14, 21]. Given the lack of thresholds specifically established for ballet dancers, it was necessary to use values from other sports. Both ball sports and dance have in common that well-established motor skills are not the main reason for success in the sport, but are necessary in order to call-up technical skills, perform well, and cope with the demands of the sport [67–70]. However, transferring the thresholds remains an interim solution until specific thresholds for the dance population are available.

58% of the ballet dancers in this cohort had holding times below average when utilizing thresholds from handball players in the Copenhagen and reversed plank [14].

Collegiate football players with less than 21 correct hand contacts in the CKCUEST have been shown to have an increased risk of injury [13]. Using this threshold, 75% of female and 38% of male dancers were unable to reach this value in this cohort. A basic level of upper body strength may be beneficial for ballet dancers. Upper body strength is exemplarily necessary to perform lifts in both males and females, as well as in transitions between positions and figures [71] . Additionally, classical dance companies are increasingly incorporating pieces with elements from other dance styles, which place greater demands on the upper extremity. However, the thresholds used for this study may not be suitable or reachable for ballet dancers, highlighting the need for ballet-specific thresholds to be established in future research.

### Limb symmetry index

In this study, a high number of dancers with asymmetries greater than 10% between their limbs was found for five muscular endurance and hop tests, with large variability between the tests, which is in line with previous studies [8, 72]. 89% of dancers in this study showed a high symmetry of the lower extremities in the figure-of-eight-hop test, in comparison to only 56.3% in the side-hop test. Even though an asymmetry of less than 10% is generally recommended for return-to-sport decisions after injury [14, 73], previous authors have questioned the universal applicability of this explicit cut-off for all strength and hop test [41]. It is not yet clear what extent of asymmetry can be tolerated in professional ballet dancers.

The present cohort revealed a higher percentage of dancers with asymmetries greater than 10% in the side-hop test and speedy jump test compared to healthy adults [37, 74]. This is concerning considering that the side-hop test distance was already reduced to 30 cm to alleviate fear of (re-)injury. The high prevalence of ankle injuries and chronic ankle instabilities reported in previous studies [75, 76] likely contributes to the fact that only 56.3% of the dancers had asymmetries of less than 11%. It has been previously reported that ankle instabilities increase the time needed to complete the 10-repetitions side-hop test [77].

In total, 20% of the dancers showed asymmetries greater than 10% in the speedy jump and heel-rise tests, which is comparable to athletes post-reconstruction of the anterior cruciate ligament [78]. This might mainly be caused by chronic or subacute injuries or pain. Dancers are accustomed to “pushing through” pain and injuries [79], which might deny adequate rehabilitation and lead to compensatory movement patterns that spare the affected body part. Moreover, it has been reported that dancers tend to develop asymmetrical leg preferences for different tasks over years of training and asymmetric loading in choreographies [19].

### Strengths and limitations

To our best knowledge, this is the first study to include FPT data from professional ballet dancers from four opera houses and state theatres, resulting in a sample of 115 participants. A subsequent analysis considering the dancers’ ranks could not be performed due to the inconsistent definition of ranks across companies and small group sizes. Future research may find it interesting to investigate potential differences in FPT results between different companies and/or ranks of the dancers.

There was no randomization of the sequence of the FPTs, which might have resulted in an accumulation of exhaustion towards the end of the screening. Instead, the FPTs were performed in a standardized order chosen to minimize negative physiological effects of exhaustion on subsequent tests. Additionally, it should be kept in mind that the time needed per screening is greatly below a dancer’s daily workload.

The maximum relevé height in the heel-rise test was determined using a hand-held mark. The reliability of this specific method is not known yet. Following Schrefl et al. [29] and Zeller et al. [50], we recommend the use of technical devices, e.g., a laser, to determine maximum height in order to increase reliability of the results.

Even though a sensor, which was previously evaluated as valid and reliable [80, 81], was used for the hop tests, some implausible values were observed in the results of the vertical-hop test. Additionally, a previous study reported a systematic overestimation of the jump height with the inertial sensor. For future studies, we suggest performing three trials instead of one, the use of the mean value for analysis, and a ballet-specific validation of jump height assessments.

The results of the side-hop test lack comparability to other studies as a 30 cm distance was used instead of the usual 40 cm. This adjustment was made after a pilot phase of the project in which many dancers faced major problems with the 40 cm distance. Reducing the distance was previously suggested by Schneider et al. at a conference [82] to reduce fear of injury in adolescent athletes and thus increase the effort to perform the maximum number of hops. Moreover, the sensor used only offered a 30 cm distance.

## Conclusion

This study presents results of a comprehensive performance screening battery using FPTs to evaluate stability, muscular endurance & strength, and hop skills in professional ballet dancers. We found low values of strength performance, high variability between individual test results of the dancers, and a high number of dancers with large limb asymmetries in this cohort. It is recommended to implement regular performance screenings in professional ballet companies to determine the status quo of both the individual dancer and the company as a whole. Based on regularly updated screening results, individualized, sex-specific and systematically planned strength & conditioning training can be implemented in the sense of performance enhancement and injury risk reduction. However, further research is needed to refine the screening battery and reduce the time required for the assessments, thereby improving feasibility of regular screenings.

## Data Availability

The data that support the findings of this study are not openly available due to reasons of sensitivity and are available from the corresponding author upon reasonable request.

## Abbreviations

FPT: Functional Performance Test
BESS: Balance Error Scoring System
CKCUEST: Closed Kinetic Chain Upper Extremity Stability Test
LSI: Limb Symmetry Index
Bpm: Beats per minute
